# Factors involved in prolonged effect of neuromuscular blockade in therapeutic hypothermia

**DOI:** 10.1186/cc13690

**Published:** 2014-03-17

**Authors:** H Tanaka, T Mochizuki, S Ode, S Ishimatsu

**Affiliations:** 1 St Lukes International Hospital, Akashi-Chou Chuo-Ku, Japan

## Introduction

Therapeutic hypothermia (TH) following out-of-hospital cardiac arrest (OHCA) improves neurological outcomes in such patients. During TH, neuromuscular blockade is used to control shivering in the patient. In our hospital, we use vecuronium as a neuromuscular blocker. However, occasionally, prolonged effects of vecuronium delay accurate evaluation of patients' neurological function or extubation. Unfortunately, the factors involved in the prolonged effect of vecuronium in TH remain unclear.

## Methods

We conducted a retrospective cohort study of patients managed with TH following OHCA at our institution from April 2010 to September 2013. We defined full-muscle reaction to train-of-four stimulation (TOF) as the end of effects of vecuronium. In this study, the time from the end of vecuronium administration to full-muscle reaction to TOF was evaluated as the outcome. We calculated the adjusted hazard ratio (HR) for the outcome using Cox regression analysis after adjustment for age, gender, albumin levels, estimated glomerular filtration rate, temperature at the end of vecuronium administration, and total amount of vecuronium per kilogram of body weight.

## Results

In total, 52 patients were evaluated in this study (86.5% male; average age, 55.2 years). The median time from the end of vecuronium administration to full-muscle reaction to TOF was 660 minutes (maximum: 10,363 minutes; minimum: 72 minutes). Blood albumin levels on admission decreased the time (adjusted HR: 2.31, 95% CI: 1.073 to 5.004; *P <*0.05). However, other factors had no significant difference on the time. The amount of vecuronium per kilogram of weight had no significant differences (adjusted HR: 1.073, 95% CI: 0.869 to 1.325; *P *= 0.511).

## Conclusion

The time from the end of vecuronium administration to full-muscle reaction to TOF decreased in relation to blood albumin levels on admission. Other factors had no significant difference on the outcome time. Taken together, the outcome time may be determined by albumin levels and not by the administered amount of vecuronium per kilogram of body weight. Based on this result, we suggest that blood albumin levels on admission should be taken into consideration for appropriate use of vecuronium in TH.
